# Research partnerships to solve health problems

**DOI:** 10.2471/BLT.16.030116

**Published:** 2016-01-01

## Abstract

The research landscape in sub-Saharan Africa has transformed over the last two decades. Charles Mgone talks to Fiona Fleck about building partnerships.

Q: How did you become interested in medicine and research?

A: I was good at biology at school and keen on science. Initially I was interested in astronomy but since there were no such courses in university – perhaps it was only a dream – I ended up studying medicine. From day one, I wanted to work with children and train as a paediatrician. I felt that adult medicine was fairly established but that there was a lot of potential for research on paediatric issues. When I started practising as a paediatrician, I discovered that there were many unanswered questions. I wondered “why are we just treating them? Why aren’t we preventing these conditions happening in the first place?” One of my early questions was on malnutrition. I wondered why children in the same family, or for example one twin, would develop marasmus and the other kwashiorkor (two different forms of malnutrition) while fed on the same diet. This didn’t happen all the time – I wonder if this question has ever been answered – but this apparent discrepancy was one of the things that fascinated me. Why does the body react to disease in a different way in certain individuals or even in identical twins? Then I got involved in sickle cell research, a problem in Africa where I lived, and looked into why children with this condition were more resistant to malaria.

Q: How did you become involved in tropical disease research?

A: After studying paediatrics, I went on to do genetics research. Initially, there were no opportunities to do tropical disease research. I started investigating porphyria, which – though rare globally – is more common in Europe including in western Scotland, where I was working, and in South Africa. I became one of the leading experts in this condition but continued to be fascinated with infectious diseases, especially tropical diseases. One day, I decided enough was enough and I left Scotland for Papua New Guinea to work on malaria. After I got there, I was working on many infectious diseases, including measles, malaria and tuberculosis and I also started concentrating on HIV infection. So in Papua New Guinea, I had gravitated from clinical medicine to pure research.

Q: Is that how you became involved in the European & Developing Countries Clinical Trials Partnership (EDCTP)? What does this organization do? 

A: The EDCTP was created in 2003 with the mission of accelerating the development of new or improved drugs, vaccines, microbicides and diagnostics against the three main poverty diseases of HIV, tuberculosis and malaria. This is taking place through a partnership between the European Union (EU) and sub-Saharan African member states in collaboration with other global partners working on these diseases, such as the private sector and funders. The EDCTP is also involved in health research capacity strengthening and developing an enabling environment to ensure that products are developed by conducting clinical trials that reflect current best practices. I was invited to join the EDCTP and so joining the organization was a natural progression, following on from my work in Papua New Guinea. After one year at the EDCTP, I was asked to head the organization and I have been doing this for the last nine years.

Q: What has the EDCTP achieved under your stewardship?

A: Ten years ago, capacity building in research was not considered cost-effective. Today that has completely changed and it is deemed to be value for money. That change in the perceptions among government decision-makers is one of our main achievements. We worked to create an environment in which local researchers could thrive, by helping to establish ethics and regulatory boards, and training people to take on the many roles needed for clinical research and the infrastructure needed to administrate it, just as research is done in Europe, North America or anywhere else in the world. Now everyone involved in the research process from funding to discovery realizes the importance of creating the right environment so that local researchers in developing countries can do the research themselves. During this 10-year period the EDCTP has funded 100 clinical trials and diagnostics studies in 24 different African countries. Our efforts to develop research capacity in sub-Saharan African countries have paid off: more than 70% of the principle investigators of these projects were Africans.

Q: What kind of partnerships did you and your team forge between developing countries and Europe?

A: We wanted to speed up the pace of discovery and innovation for public health, but we knew this could not happen without partners in European countries. At the same time, we didn’t want Northern partners parachuting into Africa, doing experiments and going away again. We wanted a genuine partnership between developing countries and their partners in the industrialized world and a sense of local ownership. We achieved that by finding scientists in developing countries to join our programme. These scientists became closely involved in the decision-making process. Now the EDCTP is a European–African partnership with African policy-makers who are closely involved in the decision-making. We also have a group of 15 African countries who are the owners of the programme and who contribute financially to the research programmes.

Q: What kinds of innovations have you helped to spur?

A: We have had many successes. We supported the clinical studies that led to WHO guidelines on prevention of mother-to-child HIV transmission during pregnancy and breastfeeding. We also supported the studies that led to the development of second-line treatment for HIV infection in Africa and the registration of paediatric formulations, which are now available in many African countries affected by the HIV epidemic. We have also done a lot of work on malaria in pregnancy. We managed to expand research programmes looking at the big three infectious diseases – HIV infection, tuberculosis and malaria – and we are now looking at neglected diseases such as schistosomiasis and leishmaniasis.

Q: How do you bring such diverse groups together?

A: We have lots of meetings. We talk to each other a lot to build trust, which is the most important thing. If you can prove to your partner that you can deliver, why separate, overlap or duplicate efforts? A good example of one of our research collaborations is a large clinical trial aimed at shortening tuberculosis drug regimens from six to four months. The trial – known as Rapid Evaluation of Moxifloxacin in Tuberculosis – took place in several countries in Africa, Asia and Latin America and African researchers recruited the majority of patients. This shows what can be achieved in global health through partnerships and international collaboration and can be used as a model for future trials. That was also a good example of how you can get value by working in a partnership based on trust.

Q: How do research projects get off the ground?

A: We start with an idea and issue a call, explaining the research project we have in mind. For example, we want to develop a vaccine against malaria through research done with partners in Europe or other industrialized countries. Often before we issue a call, we find interested partners by holding a stakeholders’ meeting in which we invite funding agencies, scientists, regulatory authorities and pharmaceutical companies to discuss the idea. They then present their papers on the possibilities, the programmes that are already in the pipeline and their policies. Based on this stakeholder meeting, we formulate the project. From time to time, we call meetings with industry to try to find out the areas in which we can collaborate in public–private partnerships. There were many questions: when can partners work with industry without making compromises, what are the drawbacks? What can you get out of the partnership? So to answer these questions we formulated a policy on how to work in partnerships with industry and others. 

Q: How do you decide on what gets researched, what are your research priorities?

A: We have an advisory committee with experts on all the diseases we are working on, as well as experts on economics, the behavioural sciences and finance. This is a kind of think-tank, and together with them we, in the secretariat, devise a strategy and business plan, for the long and medium term. This strategy is based on our mission to work on diseases of poverty, such as malaria and other neglected tropical diseases, plus any other diseases that are not receiving enough attention from industry because of market failure. Based on our mission this think-tank looks at a five-year programme. Currently we are working on annual work plans with funding from EU member states and the EU. This strategy is based on portfolios and, on the advice from these experts, we review what are the projects, what are the needs, how can we achieve them and which partners will be required. It’s a well thought-through process which keeps on being developed and with the landscape analysis, things change. For instance, I recall when we were working on microbicides, there were quite a lot of failures in the field, the thinking in scientific communities was that we needed to do more background research, the changing landscape meant that we had to keep updating our strategy and work plans.

Q: How has the EDCTP changed over the time you have been in charge?

A: We started with a focus on HIV, malaria and tuberculosis and have broadened out to other neglected infectious diseases as well. We started with 15 EU member states and now there are many more European and African states. The Partnership has also changed its legal status from a European Economic Interest Group, an international association, in which countries in sub-Saharan Africa and Europe are the members. By members, we mean they are engaged in policy- and decision-making, but any country – not just members – can receive funding for research projects, in accordance with the Horizon 2020 guidelines that govern applications for EU funding. 

Q: What have been the challenges at the helm of the partnership?

A: It was a challenge in the beginning to bring together all the European member states so that they could work as one large consortium and in partnership with developing countries in particular sub-Saharan Africa. At the same time we had to bring in the private sector – the pharmaceutical companies – and philanthropic partners. In the end, they pooled their funds and scientific questions together and our secretariat helped to bring them together to form a genuine partnership with African countries, and not one of giver and receiver. 

